# Circulating GLAST^+^ EVs are increased in amyotrophic lateral sclerosis

**DOI:** 10.3389/fmolb.2024.1507498

**Published:** 2024-11-21

**Authors:** Davide Raineri, Fabiola De Marchi, Beatrice Vilardo, Camilla Barbero Mazzucca, Lorenza Scotti, Natasa Kustrimovic, Letizia Mazzini, Giuseppe Cappellano, Annalisa Chiocchetti

**Affiliations:** ^1^ Department of Health Sciences, Interdisciplinary Research Center of Autoimmune Diseases-IRCAD, Università del Piemonte Orientale, Novara, Italy; ^2^ Center for Translational Research on Autoimmune and Allergic Diseases, University of Piemonte Orientale, Novara, Italy; ^3^ ALS Center, Azienda Ospedaliero-Universitaria Maggiore della Carità, Novara, Italy; ^4^ Department of Neurology, University of Piemonte Orientale, Novara, Italy; ^5^ Department of Translational Medicine, University of Piemonte Orientale, Novara, Italy

**Keywords:** extracellular vesicles, amyotrophic lateral sclerosis, glutamate transporter, biomarker, flow cytometry

## Abstract

Amyotrophic lateral sclerosis (ALS) is a neurodegenerative disorder, hallmarked by the gradual deterioration of motor neurons, culminating in muscle weakness and fatal paralysis. The exact etiology of ALS remains elusive, and there is a critical need for reliable biomarkers to aid in diagnosis and monitoring of disease progression. Extracellular vesicles (EVs) have emerged as promising candidates for biomarker discovery in neurodegenerative diseases such as ALS, giving access to pathologically relevant tissues otherwise typically challenging or invasive to sample. Indeed, EVs can derive by many cell types within the central nervous system, cross the blood-brain barrier and reach the blood, where they can be easily measured. One of the central mechanisms implicated in ALS pathology is glutamate excitotoxicity, which involves excessive glutamate accumulation due to impaired uptake by astrocytes and other glial cells, leading to neuronal damage. GLAST is a key glutamate transporter responsible for maintaining extracellular gluta-mate levels, and its dysregulation is thought to contribute significantly to ALS development and associated neuropathogenesis. Here, we applied a quick and validated method, to evaluate GLAST^+^ EVs in ALS patients’ plasma and age-matched healthy controls. We found an increase in GLAST^+^ EVs that holds promise for uncovering novel diagnostic and therapeutic avenues in ALS research.

## 1 Introduction

Amyotrophic lateral sclerosis (ALS) is a neurodegenerative disorder characterized by the progressive degeneration of motor neurons in the brain and spinal cord (Rojas et al., 2020). ALS leads to muscle weakness, paralysis, and ultimately respiratory failure, with a fatal outcome of the disease within 3–5 years from symptom onset (Wijesekera and Leigh, 2009). Despite extensive research efforts, the exact etiology of ALS remains elusive, and there is a critical need for reliable biomarkers to aid in early diagnosis and monitoring of disease progression. Extracellular vesicles (EVs) have gained significant attention in the field of ALS research as potential carriers of diagnostic biomarkers (Barbo and Ravnik-Glavač, 2023), since in the brain they are released by various cell types, both neuronal and non-neuronal, including astrocytes, neurons and glial cells (Schnatz et al., 2021). Size and mechanisms of biogenesis are the conventional classification approaches for EVs (Théry et al., 2018). Exosomes (30–150 nm in diameter) are derived from endosomes released from multivesicular bodies (MVBs) after fusion with the plasma membrane. In contrast, microvesicles (also termed ectosomes, 100–1,000 nm in diameter) are larger EVs generated by direct shedding from the plasma membrane. CD9, CD63 and CD81 are major members of the tetraspanin family frequently used as EVs markers. However, tetraspanins are not equally expressed in all EVs, but rather show heterogeneity that reflects the expression levels in their secretory cells (Kugeratski et al., 2021).

In human body, the most relevant reservoir of EVs is the bloodstream. Within this milieu, the most abundant EVs are originated from resident cells, namely, leukocytes, endothelial cells, and platelets (Alberro et al., 2021). Among these, EVs derived from platelets may play a crucial role in modulating optimal brain function and serve as a significant reservoir of neurotransmitters, hence potentially facilitating intercellular communication and contributing to the regulation of neural activity (Leiter and Walker, 2020). Upon activation, platelets release EVs that encapsulate a variety of bioactive molecules, carrying them to distant cellular recipients, that are otherwise inaccessible to the platelets themselves. This capacity underscores the emerging role of EVs as critical mediators in facilitating cell-to-cell communication across substantial distances, thereby coordinating intercellular interactions within the body (Maione et al., 2020).

In ALS pathology, glutamate, which is the primary excitatory neurotransmitter in central nervous system (CNS), plays a pivotal role in maintaining neuronal health and function. Its extracellular concentration is intricately regulated by astrocytic glutamate transporters, specifically known as excitatory amino acid transporter 1 (EAAT1 or GLAST or GLT-1). Dysregulation of GLAST has been associated with neuronal death (Pajarillo et al., 2019). Interestingly, Silverman et al. showed increased concentration of astrocyte-derived EVs, expressing GLAST on their surface, in the brain and spinal cord of SOD1^G93A^ ALS mice (Silverman et al., 2019). However, as of today, no data regarding the presence of GLAST^+^ EVs in humans are available.

Given the significance potential of EVs as biomarkers, their identification is challenging due to the lack of appropriate methods. Current ones (i.e., ultracentrifugation and ultrafiltration, among the others) necessitate sample manipulation which may represent a stressing condition that can promote the release of EVs during processing and/or induce cell damage (Zhao et al., 2021).

In the present study, we have applied and adapted a commercially available flow cytometric kit, which does require neither EVs isolation nor extensive sample manipulation and it has been validated in our laboratory (Cappellano et al., 2021; Raineri et al., 2022) and by others (Marchisio et al., 2020), to type the most abundant EVs (platelet-, endothelial- and leukocyte-derived EVs) on plasma of ALS patients. As a proof of concept of the existence of GLAST^+^ EVs, we refined this method to detect within the plasma of ALS patient’s astrocyte derived-EVs expressing GLAST marker on their surface.

Our findings showed that GLAST^+^ EVs are increased in ALS patients compared to age-matched healthy control. No discernible association with clinical data from ALS patients was identified.

## 2 Materials and methods

### 2.1 Study design

Peripheral venous blood samples were collected from ALS patients (n = 61) and healthy age and sex matched controls, HC (n = 30). Subjects were recruited at the Regional Expert ALS Center (CRESLA) at the Neurology Clinic of the “Maggiore della Carità” University Hospital in Novara. All enrolled subjects provided written informed consent to participate in the study. The ethical committee has been approved (CE n. 184/20). Inclusion and exclusion criteria for patients are summarized in the supplementary material. After enrollment, subjects were assessed and monitored throughout the course of their disease until the terminal stage using the Amyotrophic Lateral Sclerosis Functional Rating (ALSFRS-R) scale, Forced Vital Capacity (FVC)%, Body Mass Index (BMI), and neurological objectivity. Monthly changes in ALSFRS-R, FVC%, and BMI between the first and last assessments were calculated using the formula: (measurement at the first assessment - measurement at the last available assessment)/(date of the first assessment - date of the last assessment). Samples were collected at the time 0 (T0).

### 2.2 Blood sampling and plasma isolation

Samples of peripheral venous blood from ALS patients and HC were collected in sodium-citrate pre-coated vials (BD Vacutainer). Blood samples was centrifuged at 3,500 rpm for 15 min and plasma was frozen at −80°C until further analysis.

### 2.3 Flow cytometry analysis of circulating EVs

A customized EVs detection kit (Becton and Dickinson, NJ, United States) was employed to characterize EVs derived from the whole blood of both patients and HC, following a previously established protocol (Cappellano et al., 2021; Raineri et al., 2022). Briefly, 0.5 μL of APC-conjugated lipophilic cationic dye (LCD) and FITC-conjugated phalloidin, along with 5 μL each of anti-CD31-PECy7, anti-CD41a-PE, and anti-CD45-BV510 were added in 184 μL of filtered phosphate buffer sulphate (PBS). Subsequently, 5 μL of whole blood was incubated with this mix. To exclude apoptotic bodies or damaged EVs, phalloidin was added to each sample. Before staining, the reagent mixture underwent centrifugation at 13,200 rpm for 15 min to prevent antibody aggregates. After incubation for 45 min at room temperature (RT), 2 mL of filtered PBS were added, and the samples were analyzed by flow cytometry (FACSymphony A5, Becton and Dickinson, NJ, United States), with the threshold set in the fluorescent channel as recommended in the literature (Mobarrez et al., 2010) allowing the identification of either exosomes and microvesicles. FACSDiva software (Becton and Dickinson, NJ, United States) was employed for flow cytometry data analysis. The calculation of EVs per microliter (EVs/μL) was determined using the following formula:

Number of EV events for a given population x dilution factoracquired volume

Instrument stability was assessed by acquiring data from four independent true count tubes, each measured three times (Becton and Dickinson, NJ, United States).

### 2.4 Flow cytometry analysis for GLAST^+^ EVs identification

GLAST^+^ EVs were detected in a volume of 50 µL of plasma from both ALS patients and HC, without the need for ultracentrifugation, utilizing the aforementioned protocol with some adaptations. Specifically, within the staining anti-CD31 and anti-CD45 monoclonal antibodies (mAbs), were replaced by 2 µL of anti-GLAST PE-conjugated antibody (Miltenyibiotec, Teterow, Germany). To exclude platelet (PLT)-derived EVs from the analysis, anti-CD41-FITC mAb was employed, whereby CD41^+^ events were excluded in the gating strategy. The isotype-PE antibody (Miltenyibiotec, Teterow, Germany) served as a control to draw the GLAST^+^ EVs region. Samples were processed using FACSymphony A5 (Becton and Dickinson, NJ, United States), and FACSDiva software (Becton and Dickinson, NJ, United States) was employed for flow cytometry data analysis. The GLAST^+^ EV count per microliter was determined utilizing the formula above.

### 2.5 Flow cytometry analysis for CD63 and CD81 tetraspanin identification

Tetraspanin were detected using the same protocol described for GLAST^+^ EVs adding 5 µL of anti-CD63 BV605-conjugated and anti-CD81 BV711-conjugated together with anti-GLAST PE-conjugated and anti-CD41 FITC-conjugated. Samples were processed using FACSymphony A5 (Becton and Dickinson, NJ, United States), and FACSDiva software (Becton and Dickinson, NJ, United States) was employed for flow cytometry data analysis.

### 2.6 Statistical analysis

Data from the flow cytometry analysis were analyzed by GraphPad Prism Software for comparisons between ALS patients and HC using non-parametric Mann-Whitney U-test. The level of significance was set at p-value < 0,05.

## 3 Results

### 3.1 Clinical characteristics

Relevant clinical data regarding enrolled patients and their stratification are summarized in Table 1.

**TABLE 1:** Clinical characteristics.

Features		N (%)
Gender	M	39 (63.93)
	F	22 (36.07)
Diabetes	Yes	4 (6.67)
	No	56 (93.33)
	Missing	1
Type of disease	Spinal	41 (67.21)
	Bulbar	20 (32.79)
Endocrine diseases	No	54 (93.1)
	Yes	4 (6.9)
	Hypothiroidism	3 (75)
	Dyslipidemia	1 (25)
	Missing	3
Mutations	C9Orf72	8 (13.11)
	KIF5A	1 (1.64)
	No	49 (80.33)
	SOD1	2 (3.28)
	TDP-43	1 (1.64)
Progression	Slow	34 (55.74)
	Fast	27 (44.26)
Cognitive impairment	Normal	39 (63.93)
	ALS-ci	20 (32.79)
	ALS-bi	1 (1.64)
	FTD	1 (1.64)
Phenotype	Classic	32 (52.46)
	Bulbar	20 (32.79)
	PUMN	6 (9.84)
	Respiratory	3 (4.92)
Parameters/Scores	Age at blood collection, average (SD)	62.88 (12.60)
	Average monthly change in FVC, median (Q1-Q3)	−1.93 (−3.78;-1.00)
	Average monthly change in BMI, median (Q1-Q3)	−0.02 (−0.16; 0.15)
	Average monthly change in ALFRSR, median (Q1-Q3)	−0.60 (−1.31;-0.27)

### 3.2 Circulating EVs profile did not differ between ALS patients and HC

**FIGURE 1:**
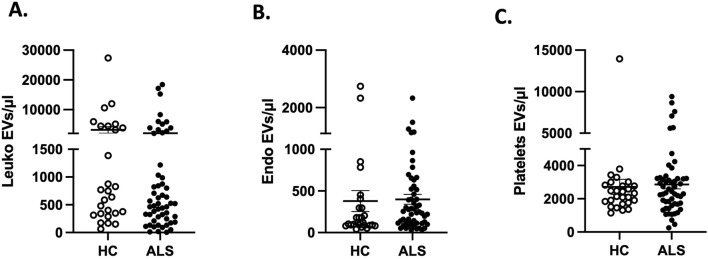
Absolute counts of **(A)** leukocytes-(leuko), **(B)** endothelial-endo and **(C)** platelets-derived EVs in ALS (n = 61) compared to HC (n = 30).

By applying flow cytometry (Cappellano et al., 2021; Raineri et al., 2022), we found that the absolute count of leukocytes-, endothelial- and PLT-derived EVs was similar in both ALS patients and HC (Figures 1–C; Supplementary Figure S1).

### 3.3 GLAST^+^ EVs counts are increased in ALS patients compared to HC

**FIGURE 2:**
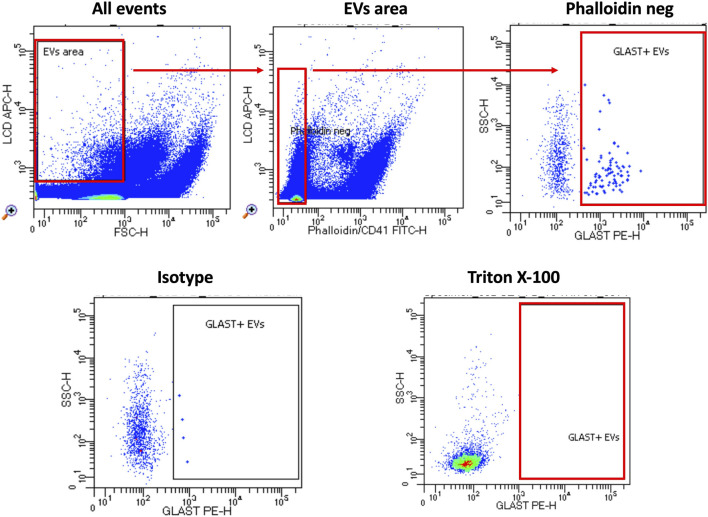
Gating strategy. The custom kit from BD incorporates an APC-emitting lipophilic cationic dye (LCD), which permeates double-layer structures assisted by membrane potential. This dye effectively stains EVs and cells, both dotated of a lipidic membrane. Additionally, Phalloidin-FITC selectively binds to the cytoskeleton protein actin, specifically targeting EVs/cells with damged membrane. To exclude platelets from the analysis, anti-CD41-FITC antibody was used. EVs were then characterized based on their LCD positivity and smaller size as measured by forward scatter (FSC) (EVs area). Within intact EVs region (defined as LCD+/phalloidin-), GLAST^+^ EVs were identified. To verify the specificity of staining, an isotype-PE antibody served as a negative control. Lastly, samples were treated with Triton X-100 solution and re-acquired in order to confirm the specificity of LCD staining for EVs. Samples were acquired using FACSymphony A5 and data were analyzed using FACSDiva software.

By modifying a commercially available kit’s method (Cappellano et al., 2021; Marchisio et al., 2020; Raineri et al., 2022), we were able to identify GLAST^+^ EVs in plasma specimens of ALS patients and HC by flow cytometry. Figure 2 shows the gating strategy in detail.

**FIGURE 3:**
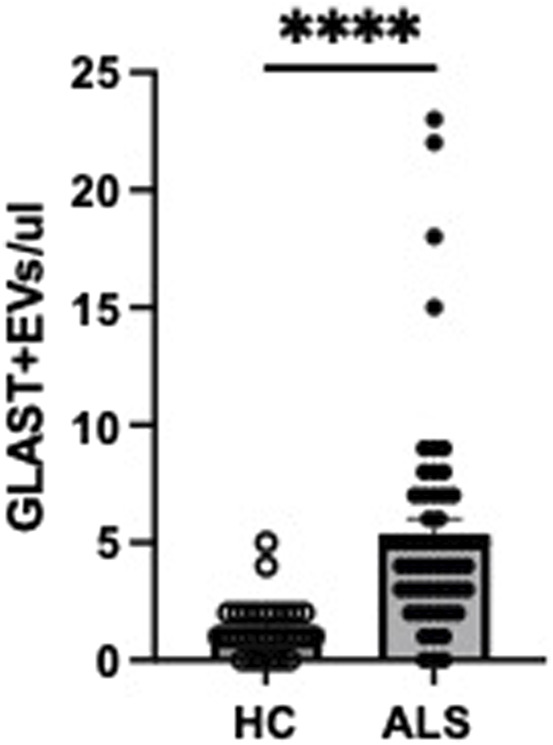
GLAST^+^ EVs counts in ALS patients (n = 61) and HC (n = 30). Mann-Whitney test was used, ****p < 0.0001.

**FIGURE 4:**
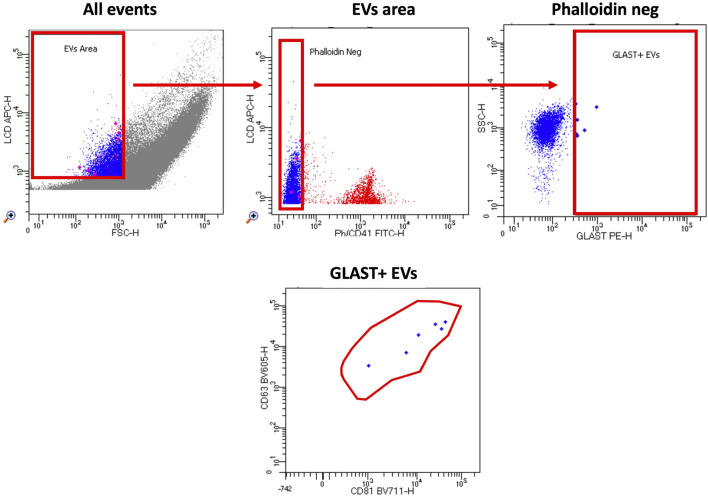
Gating strategy. The custom kit from BD incorporates an APC-emitting lipophilic cationic dye (LCD), which permeates double-layer structures assisted by membrane potential. This dye effectively stains EVs and cells, both dotated of a lipidic membrane. Additionally, Phalloidin-FITC selectively binds to the cytoskeleton protein actin, specifically targeting EVs/cells with damged membrane. To exclude platelets from the analysis, anti-CD41-FITC antibody was used. EVs were then characterized based on their LCD positivity and smaller size as measured by forward scatter (FSC) (EVs area). Within intact EVs region (defined as LCD+/phalloidin-), GLAST^+^ EVs were identified. Finally, tetraspanin positive events were identified according to the expression of CD63 and CD81 in BV605 and BV711, respectively.

Absolute counts of GLAST^+^ EVs were found to be significantly increased in ALS patients compared to HC (Figure 3). To confirm that the events evaluated as GLAST^+^ were really EVs, co-expression of tetraspanins was evaluated (CD63 and CD81). As shown in Figure 4, all GLAST^+^ events were also positive for both the evaluated tetraspanins. Lastly, the correlation between all EVs counts with clinical data of ALS patients, that included mutations, form of the disease, phenotype, cognitive profile, and progression rate (fast or slow), as well as non-categorical clinical variables, including delta ALSFRS-R, delta BMI, and delta FVC was performed. No significant correlations were observed among all the variables considered, as shown in the Supplementary tables.

## 4 Discussion

The identification of EVs in biological fluids presents a challenge due to the lack of standardized methods. Nowadays the principal requirements for diagnostic methods are to be quick and reliable, criteria that many existing methods often fail to meet. In this study, we introduce a modified method for cell-specific EVs identification that offers both speed and reliability. This method has been rigorously tested and validated in independent laboratories, comprising Lanuti’s one (Marchisio et al., 2020) and ours (Cappellano et al., 2021; Raineri et al., 2022). This method allows the identification of a range of EVs from 100–1,000 nm, including either microvesicles (MVs) (around 95%) and exosomes (around 5%) (Marchisio et al., 2020).

In ALS neuroinflammation involves the activation of immune cells and release of inflammatory molecules within the CNS. This inflammatory response contributes to the progressive degeneration of motor neurons characteristic of ALS. Interestingly, neuroinflammation can also influence peripheral blood components, including platelets. In ALS, neuroinflammation can lead to alterations in platelet function and activation. Studies have suggested that platelets in ALS patients may exhibit abnormal activation states, release pro-inflammatory factors, and interact with immune cells, potentially exacerbating neuroinflammation and neuronal damage (Leiter and Walker, 2020). Additionally, platelets serve as a primary reservoir of serotonin and several other neurotransmitters including γ-aminobutyric acid (GABA), dopamine and glutamate. All these neurotransmitters can be found as cargo of platelet derived EVs and are essential for the intercellular communication between brain cells. Studies have demonstrated significantly reduced platelet serotonin levels in ALS patients, which positively correlate with patient survival (Dupuis et al., 2010).

As of today, there is no clear consensus on the numerical differences in EVs between ALS patients and HC. Some authors have reported increased concentration and size of EVs in the plasma of ALS patients (Sproviero et al., 2019), while other reported no numerical variation in number of EVs between ALS patients and HC, whether the evaluation of EVs have been performed in plasma (Sproviero et al., 2018), cerebrospinal fluid (CSF) (Thompson et al., 2020) or serum (Lo et al., 2021). Interestingly, Lo et al. have evaluated the number of EVs isolated from human frontal cortex, spinal cord, and serum samples of ALS and HC demonstrating that numbers of EVs did not significantly differ between two cohorts, regardless of tissue type (Provenzano et al., 2023).

In our study, we observed similar counts of PLT-EVs in ALS patients compared to HC. Additionally, no differences were found in the counts of leukocyte-derived and endothelial-derived EVs between the two groups thus aligning our results to those of several other groups. Variations between studies, particularly among control participants, may arise from differences in isolation and size determination methodologies, as well as various biological factors such as age, sex, tissue of origin, cargo composition, time of sample collection, EVs phenotype, and disease status. Additionally, we believe that part of the discrepancy with our data might be due to the different method applied to identify leukocytes-derived EVs. Indeed, we analyze only integer EVs, while other methods use a standardized calibrated-bead strategy, using polystyrene beads (Megamix-Plus, Bio-Cytex, France), that lacks the capability to discriminate between intact and damaged EVs.

Our study’s primary finding highlights a significant increase in GLAST^+^ EV levels in ALS patients compared to HC. Our experimental conditions impede to rule out if the identified GLAST^+^ EVs are exosomes or ectosome, since the method used to identify EVs only detects vesicles bigger that 100 nm. Moreover, understanding the biological origin and functional effects of EVs subtypes is challenging due to the moderate differences in their physical properties and the absence of reliable markers. Exosomes are composed of endosomal sorting proteins required for transport (ESCRT) and tetraspanins (Jeppesen et al., 2019). In contrast, ectosomes are enriched in cytoskeletal proteins, glycolytic enzymes, integrins and annexins but may also express tetraspanins. Nevertheless, for the purpose of this study, the exact biogenesis of these EVs is beyond the scope.

Glutamate excitotoxicity, a process implicated in ALS pathology (Provenzano et al., 2023), is closely tied to our findings. GLAST is responsible for synaptic glutamate clearance thus playing a crucial role in maintenance of optimal extracellular glutamate levels. This crucial function prevents the accumulation of glutamate in the synaptic cleft, mitigating the risk of excitotoxicity. Dysregulation of GLAST function may play a significant role in excitotoxicity and its associated neuropathogenesis, as it occurs in ALS (Silverman et al., 2019). Notably, Silverman et al. demonstrated GLAST^+^ EVs in the brain and spinal cord of SOD1^G93A^ ALS mice, suggesting a potential link between GLAST^+^ EVs and ALS pathogenesis (Silverman et al., 2019). Indeed, EVs from brains and spinal cords of the SOD1^G93A^ ALS mouse model, as well as from the spinal cords of human familial ALS patients with SOD1 mutations, contained abundant misfolded, non-native, disulfide-cross-linked aggregated SOD1 (Silverman et al., 2019). One of the mechanism responsible of protein aggregation maybe mediated by the heparan sulfate (HS) chains, essential components of the extracellular matrix (ECM) and cell surface proteoglycans, that are cleaved by heparinase; this is of particular interest in the context of neurodegenerative diseases such as Alzheimer’s disease, where HS-rich Aβ deposits are commonly formed (Snow et al., 2021), and Parkinson’s disease, in which HS are present in Lewy bodies (Cohlberg et al., 2002). Heparinase also plays a critical role in autophagy (Shteingauz et al., 2015) and in the exosome generation by modulating the structural integrity of ECM promoting the release of exosomes from cells (Ramani et al., 2013). Autophagy and lipid rafts together facilitate the selective sorting, formation, and release of EVs. Lipid rafts provide the structural basis for EV membrane curvature and stability, while autophagy enables selective cargo packaging and recycling processes, ensuring that EVs serve specific signaling functions in intercellular communication (Pollet et al., 2018; Zubkova et al., 2024). Interestingly, it has been shown that heparinase inhibitor blocks autophagy leading to induction of apoptosis in glioblastoma cells (Manganelli et al., 2023). Given this background, although the potential role of HS in ALS pathogenesis warrants further investigation, we can hypothesize that a similar mechanism involving HS and protein aggregation could be at play also in ALS. This may open a new avenue for ALS treatment.

In humans, prior research has shown GLAST protein loss in the motor cortex and spinal cord of ALS patients, along with abnormal glutamate metabolism (Bristol and Rothstein, 1996; Rothstein et al., 1995; Sugiyama et al., 2017).

While our study did not find a clear association of GLAST^+^ EVs with any evaluated clinical parameter, we hypothesize that these EVs may influence glutamate excitotoxicity once released by astrocytes, potentially impacting neuronal viability (Rothstein et al., 1995).

This study is subject to several limitations that necessitate consideration. Firstly, we acknowledge the absence of a longitudinal evaluation of GLAST^+^ EVs in the course of the disease. Moreover, additional cohorts comprising patients with various neurodegenerative disorders. The inclusion of such cohorts would allow for a comprehensive assessment of the specificity of GLAST^+^ EVs in facilitating a differential diagnosis of ALS from these diseases. Future research endeavors should prioritize exploring the role of GLAST^+^ in other neurodegenerative conditions, such as Parkinson’s disease, Alzheimer’s disease, Huntington’s disease, and Frontotemporal Dementia.

Secondly, the protein cargo of GLAST^+^ EVs remains a topic of interest that warrant further investigation. These vesicles may contain additional biomarkers or molecular constituents that could offer valuable insights into the pathogenesis of ALS.

Lastly, functional studies aimed at understanding the precise impact of GLAST^+^ EVs on cellular processes associated with ALS, could contribute significantly to the understanding of the disease.

The absence of reliable markers for ALS diagnosis, prognosis, and therapeutic assessment underscores the urgent need for innovative approaches. EVs have emerged as promising candidates for liquid biopsies, given their ability to transport disease-related molecules even in complex biological environments and given the access to pathologically relevant tissues otherwise typically challenging or invasive to sample. Here, we have introduced a modified method for cell-specific EVs, demonstrating the presence of EVs expressing GLAST in healthy human blood, and their increased levels in ALS patients. Neuroinflammation, a hallmark of ALS, impacts systemic biology beyond the CNS, including the release of EVs. The increased levels of GLAST^+^ EVs in ALS patients suggest a potential link between neuroinflammation and dysregulated glutamate metabolism in ALS pathology. Despite limitations, our study underscores the potential of EVs as reservoirs of biomarkers for ALS and other diseases. The discovery of increased levels of GLAST^+^ EVs in ALS patients in our study represents a novel avenue for further investigation and offers significant potential for the development of new diagnostic and therapeutic strategies in ALS research.

## Data Availability

The original contributions presented in the study are included in the article/Supplementary Material, further inquiries can be directed to the corresponding author.
