# Supplementary material for: Circulating GLAST+ EVs are increased in amyotrophic lateral sclerosis
Source: Front Mol Biosci. 2024 Nov 21;11:1507498. doi: 10.3389/fmolb.2024.1507498 (PMC11617857; doi:10.3389/fmolb.2024.1507498)
Supplement: Supplementary file 1 [file Image1.pdf]

Supplementary Material

1 Supplementary Figures and Tables

1.1 Supplementary Figures

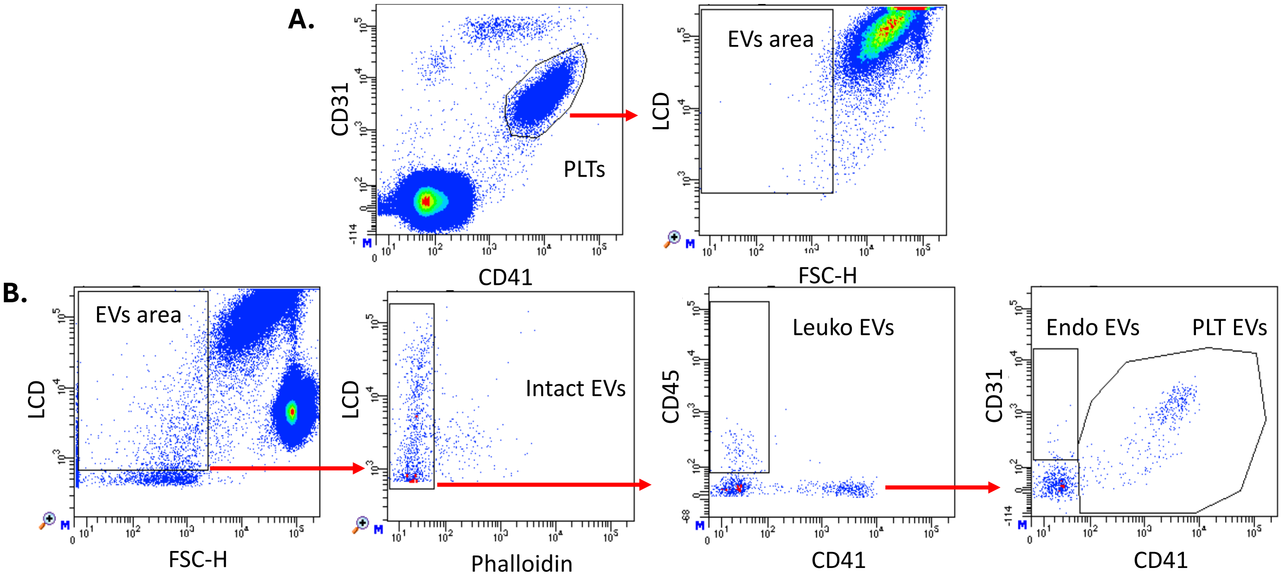

Supplementary Figure 1. *Gating strategy for the identification of leuko- platelets- endothelial-EVs. A) Platelets were identified as CD41 and CD31 double positive and they are used as dimensional reference to draw the EVs area on FSC and LCD dot plot. B) EVs were identified in the EVs area (LCD vs FSC) and the integer EVs were gated as phalloidin negative events. Finally, leukocytes-EVs (CD45+) were identified (CD45 vs CD41), then, the endothelial- (CD31+) and platelets- (CD31+/CD41+) EVs were gated among the CD45 negative EVs.*
